# *In vitro* antimicrobial activity of ethanolic fractions of *Cryptolepis sanguinolenta*

**DOI:** 10.1186/1476-0711-11-16

**Published:** 2012-06-18

**Authors:** Felix C Mills-Robertson, Samuel C K Tay, Goerge Duker-Eshun, Williams Walana, Kingsley Badu

**Affiliations:** 1 Department of Microbiology, Centre for Scientific Research into Plant Medicine, Mampong-Akwapim, Ghana; 2 Department of Clinical Microbiology, School of Medical Sciences, Kwame Nkrumah University of Science and Technology, Kumasi, Ghana; 3 Kenya Medical Research Institute Center for Global Health, Research Climate and Human Health Research Unit, Kisumu, Kenya

## Abstract

**Background:**

Following claims that some plants have antimicrobial activities against infectious microbes, the *in vitro* antimicrobial activities of different solvent fractions of ethanolic extract of *Cryptolepis sanguinolenta* were evaluated against eight standard bacteria and clinical isolates.

**Methods:**

The solvent partitioning protocol involving ethanol, petroleum ether, chloroform, ethyl acetate and water, was used to extract various fractions of dried pulverized *Cryptolepis sanguinolenta* roots. Qualitative phyto-constituents screening was performed on the ethanol extract, chloroform fraction and the water fraction. The Kirby Bauer disk diffusion method was employed to ascertain the antibiogram of the test organisms while the agar diffusion method was used to investigate the antimicrobial properties of the crude plant extracts. The microplate dilution method aided in finding the MICs while the MBCs were obtained by the method of Nester and friends. The SPSS 16.0 version was used to analyze the percentages of inhibitions and bactericidal activities.

**Results:**

The phytochemical screening revealed the presence of alkaloids, reducing sugars, polyuronides, anthocyanosides and triterpenes. The ethanol extract inhibited 5 out of 8 (62.5%) of the standard organisms and 6 out of 8 (75%) clinical isolates. The petroleum ether fraction inhibited 4 out of 8 (50%) of the standard microbes and 1 out of 8 (12.5%) clinical isolates. It was also observed that the chloroform fraction inhibited the growth of all the organisms (100%). Average inhibition zones of 14.0 ± 1.0 mm to 24.67 ± 0.58 mm was seen in the ethyl acetate fraction which halted the growth of 3 (37.5%) of the standard organisms. Inhibition of 7 (87.5%) of standard strains and 6 (75%) of clinical isolates were observed in the water fraction. The chloroform fraction exhibited bactericidal activity against all the test organisms while the remaining fractions showed varying degrees of bacteriostatic activity.

**Conclusion:**

The study confirmed that fractions of *Cryptolepis sanguinolenta* have antimicrobial activity. The chloroform fraction had the highest activity, followed by water, ethanol, petroleum ether and ethyl acetate respectively. Only the chloroform fraction exhibited bactericidal activity and further investigations are needed to ascertain its safety and prospects of drug development.

## Introduction

*Cryptolepis sanguinolenta* (Lindl.) Schltr. (Periplocaceae) is a plant mostly found in the tropical rain forest regions of Africa with several species. C *sanguinolenta* is the most common in Ghana. This species is found on mountainous territories in Ghana, especially the Akwapim and Kwahu mountains [1,2]. Paulo and Houghton [3], described the plant as a slender thin stemmed climbing shrub with orange-coloured juice in the cut stem and like most medicinal herbs or plants, the exact history on the usage of the plant is not well established, but it is confirmed that some indigenous inhabitants in the Akwapim and Kwahu mountainous areas in Ghana use the plant to manage various forms of fever, malaria and some infections caused by bacteria [4]. It has also been established that, the extract of *C*. *sanguinolenta* has antimuscarinic, vasodilating, noradrenergic, antithrombotic, anti-inflammatory, and hypoglycemic activities [5]. The part of the plant mostly used is the root and the extract is obtained in the aqueous form by boiling, or by alcohol extraction, popularly referred to as “bitters” in Ghana. Thus, *C*. *sanguinolenta* is a potential medicinal plant that must be investigated to establish its antimicrobial activity. Previous *in vitro* studies have compared the effect of ethanol, cold and hot aqueous extracts of *C*. *sanguinolenta* as antimicrobial agents using Gram positive and Gram negative organisms as well as *C. albicans*. Eighty five percent of the test microbes were inhibited by the ethanol extract while the cold and hot aqueous extracts inhibited seventy five percent of the test microbes respectively [6]. The current study investigated the antimicrobial activity of various solvent fractions of ethanolic extract of *C*. *sanguinolenta*, with the aim of identifying the bioactive fractions of the extract as well as finding out the degree of activity against selected pathogenic bacteria.

## Methodology

### Ethical issues

The study protocol was approved by the Committee on Human Research Publication and Ethics of the School of Medical Sciences, Kwame Nkrumah University of Science and Technology (CHRPE/187/10). Informed consent was obtained from participants.

### Experimental materials

Voucher specimens (Voucher # CSRPM 1911), of the roots of *C*. *sanguinolenta* were harvested, dried and stored in the herbarium of the institute until needed.

### Test organisms used

The test organisms used in this study were obtained from the Komfo Anokye Teaching Hospital (KATH), Kumasi, Ghana. These organisms were confirmed at the CSRPM using biochemical and Analytical Profile Index (API) kits. The isolates used consisted of one strain each of *Staphylococcus aureus, Staphylococcus saprophyticus, Escherichia coli, Klebsiella pneumoniae, Proteus mirabilis, Pseudomonas aeruginosa, Salmonella typhi* and *Salmonella typhimurium.* The following standard strains of bacteria were used; *S. aureus* ATCC 25923*, S. saprophyticus* ATCC 15305*, E. coli* ATCC 25922*, K. pneumoniae* ATCC 33495*, Proteus mirabilis* ATCC 49565*, P. aeruginosa* ATCC 27853*, S. typhi* ATCC 19430 and *S. typhimurium* ATCC 14028.

### Antibiotic susceptibility testing

All the isolates were subjected to antimicrobial susceptibility test using Kirby-Bauer disc diffusion method as described by NCCLS [7]. Briefly peptone water subcultures which had attained the 0.5 McFarland turbidity standards were used to seed the surface of the Muellar-Hinton agar plates. Antibiotics disks [Amikacin (30 μg/disc), Ampicillin (10 μg/disc), Penicillin (10 μg/disc), Cloxacillin (5 μg/disc), Erythromycin (15 μg/disc), Tetracycline (30 μg/disc), Gentamicin (10 μg/disc), Cotrimoxazole (25 μg/disc), Chloramphenicol (30 μg/disc), and Cefixime (30 μg/disc), Cefuroxime (30 μg/disc), and Cefotaxime (30 μg/disc)] were carefully placed on the surface of the plates and incubated at 37°C for 16–18 hours.

### Ethanolic extract *C. Sanguinolenta*

One kilogram (1 kg) of dried pulverized *C. sanguinolenta* roots was macerated in 8 L of 70% ethanol in water and stored at room temperature for 48 hours. The resultant extract was filtered, concentrated and subsequently freeze-dried. The freeze-dried sample was kept in the refrigerator at 4°C until needed. About 6 grams of the freeze-dried sample was reserved whilst the remaining was subjected to the solvent partitioning protocol as previously described [8,9].

### Phytochemical screening of the extracts

The phytochemical constituents of the ethanol extracts, chloroform fraction and the water fraction were determined. The phytochemical parameters assayed for, included saponins, reducing sugars, polyuronides, cyanogenic glycoside, alkaloid, triterpenes, phytosterols, flavonoids, anthocyanosides and phenolics [10].

### Antimicrobial activity of the fractions/compounds

The agar diffusion method was used to investigate the antibacterial properties of both the crude ethanolic extracts and sequential solvent fractions of *C. sanguinolenta*, as described in the National Committee for Clinical Laboratory Standards [11] and the National Center for Infectious Disease, Center for Disease Control and Prevention [12].

### Determination of the minimum inhibitory concentration (MIC)

The MIC values of the crude extract were determined using the microplate dilution method as described by Eloff [13]. Briefly, 100 μl of 32 mg/ml of the ethanolic extract was added to 100 μl of sterile bacteriological peptone in the first well in the 96-well microplate and mixed well with a micropipette, 100 μl of this dilution was transferred to the bacteriological peptone in subsequent wells yielding two-fold serial dilution in the original extract. The process was repeated for the other plant extracts in other columns of the microplate. A reference solution of chloramphenicol was also serially diluted in another column of the microplate as a positive control. 100 μl of actively growing test organisms (0.5 McFarland standards) was added to each of the wells except the negative control. Triplicate of each microplate was made and the procedure repeated for the other organisms. The microplates were incubated at 37°C for 24 hours. After the incubation, 40 μl of 0.2 mg/ml INT was added to each of the wells. The microplates were then examined after additional 60 minutes incubation. Bacterial growth is indicated by a red colour (conversion of the INT to formazan), and the lowest concentration at which the red colour is apparently invisible compared to the next dilution was taken as the MIC value.

### Determination of the minimum bactericidal concentration (MBC)

The MBC values were deduced from those wells with the lowest concentrations at which no growth (colour development) was observed after culturing for 24 hours of incubation as described by Nester *et al*., [14].

## Results

### Phytochemical screening

Phytochemical screening performed on the ethanol extract as well as the water and chloroform fractions revealed the presence of reducing sugars, polyuronides, alkaloids and anthocyanosides. The water fraction in addition contains triterpenes (Table 1).

**Table 1:** Phyto-constituents of the ethanol extract and the partitioned fractions

**Phytochemical parameters**	**Extract/Fraction**		
	**Ethanol**	**Water**	**Chloroform**
Saponins	-	-	-
Reducing sugars	+	+	+
Polyuronides	+	+	+
Cyanogenic glycosides	-	-	-
Alkaloids	+	+	+
Triterpenes	-	+	-
Phytosterols	-	-	-
Flavonoides	-	-	-
Anthocyanosides	+	+	+

+ = present, - = absent.

### Antibiotic susceptibility tests

**Figure 1:**
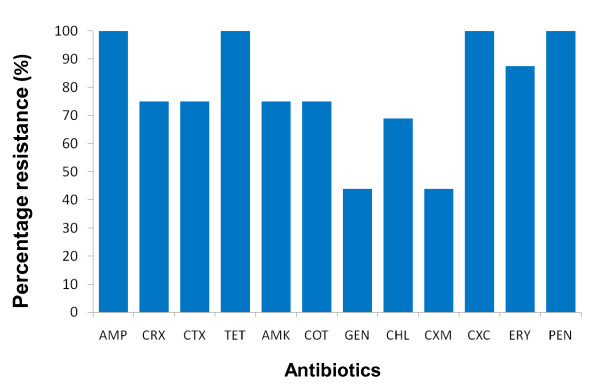
**Anti biotic*resistance patterns of micro*o*rgan*isms used.**

The antibiotic susceptibility test performed on all the 16 pathogenic bacteria revealed a high resistance pattern to all the first line antibiotics used in the study. It was observed that, all the microbes were resistant to AMP, CXC, TET and PEN, while the remaining antibiotics exhibited varying degrees of resistance and susceptibility (Figure 1).

### Susceptibility of the microbes to various fractions of *C. Sanguinolenta*

As depicted in Table 2, 11 out of the 16 (68.75%) microbes used were inhibited by the ethanol extract with average zones of inhibition ranging from 9.33 ± 0.58 to 35.67 ± 0.58 mm. Another 11 out of 16 isolates were not susceptible to the petroleum ether fraction; however, both the standard and wild strains of *Proteus mirabilis* were susceptible with 27.00 ± 1.00 mm and 14.67 ± 0.58 mm as the average zones of inhibition respectively. The chloroform fraction registered 100% inhibitory activity against all the sixteen isolates with inhibition zones averagely ranging between 9.33 ± 0.58 and 32.33 ± 0.58 mm. The ethyl acetate fraction inhibited the growth of 3 out of the 16 (18.75%) isolates used with average zones of inhibition ranging from 14.00 ± 1.00 to 24.67 ± 0.58 mm. The water fraction of *C*. *sanguinolenta* inhibited 12 out of the 16 (75.00%) microbes used with average zone diameters ranging from 8.67 ± 0.58 to 38.33 ± 0.58 mm (Table 2). Chloramphenicol inhibited the growth of *Proteus mirabilis* (ATCC 49565), *E. coli* (ATCC 25922), *S. aureus* (ATCC 25923) and *S. saprophyticus* (ATCC 15305) representing 25.00% of the total number of microbes used.

**Table 2:** **Susceptibility of microbes to the extract and fractions of*C. sanguinolenta***

**Test Organisms**	**Ethanol extract**	**Petroleum ether fraction**	**Chloroform fraction**	**Ethyl acetate fraction**	**Water fraction**	**Chloram-phenicol**
*Salmonella typhi* ATCC 19430	18.00 ± 0.00	0.00 ± 0.00	19.67 ± 0.58	0.00 ± 0.00	16.67 ± 1.15	0.00 ± 0.00
*Salmonella typhi*	0.00 ± 0.00	0.00 ± 0.00	11.33 ± 0.58	0.00 ± 0.00	0.00 ± 0.00	0.00 ± 0.00
*Salmonella typhimurium* ATCC 14028	0.00 ± 0.00	0.00 ± 0.00	11.33 ± 0.58	0.00 ± 0.00	0.00 ± 0.00	0.00 ± 0.00
*Salmonella typhimurium*	0.00 ± 0.00	0.00 ± 0.00	9.33 ± 0.58	0.00 ± 0.00	0.00 ± 0.00	0.00 ± 0.00
*Proteus mirabilis* ATCC 49565	9.33 ± 0.58	27.00 ± 1.00	20.67 ± 0.58	14.00 ± 1.00	10.33 ± 0.58	14.67 ± 0.58
*Proteus mirabilis*	20.67 ± 0.58	14.67 ± 0.58	19.33 ± 0.58	0.00 ± 0.00	18.67 ± 0.58	0.00 ± 0.00
*Pseudomonas aeruginosa* ATCC 27853	0.00 ± 0.00	0.00 ± 0.00	11.00 ± 1.00	0.00 ± 0.00	9.67 ± 0.58	0.00 ± 0.00
*Pseudomonas aeruginosa*	20.00±	0.00 ± 0.00	15.67 ± 0.58	0.00 ± 0.00	24.33 ± 1.15	0.00 ± 0.00
*Klebsiella pneumoniae* ATCC 33495	0.00 ± 0.00	0.00 ± 0.00	10.67 ± 0.58	0.00 ± 0.00	0.00 ± 0.00	0.00 ± 0.00
*Klebsiella pneumonia*	22.33 ± 0.58	0.00 ± 0.00	15.67 ± 0.58	0.00 ± 0.00	23.67 ± 0.58	0.00 ± 0.00
*Escherichia coli* ATCC 25922	19.67 ± 0.58	19.670.58	14.67 ± 0.58	0.00 ± 0.00	21.33 ± 1.15	14.33 ± 0.58
*Escherichia coli*	9.67 ± 0.58	0.00 ± 0.00	11.33 ± 0.58	0.00 ± 0.00	8.67 ± 0.58	0.00 ± 0.00
*Staphylococcus aureus* ATCC 25923	35.67 ± 0.58	32.670.58	32.33 ± 0.58	24.67 ± 0.58	38.33 ± 0.58	26.00 ± 1.00
*Staphylococcus aureus*	18.67 ± 0.58	0.00 ± 0.00	14.33 ± 0.58	0.00 ± 0.00	17.00 ± 2.00	0.00 ± 0.00
*Staphylococcus saprophyticus* ATCC 15305	20.33 ± 0.58	24.33 ± 1.15	19.67 ± 0.58	17.33 ± 0.58	21.00 ± 1.00	19.33 ± 0.58
*Staphylococcus saprophyticus*	10.00 ± 0.00	0.00 ± 0.00	10.67 ± 0.58	0.00 ± 0.00	9.33 ± 0.58	0.00 ± 0.00

### MICs and MBCs of the ethanol extract of *C*. *Sanguinolenta* and its partitioned fractions

The MICs and MBCs of the partitioned fractions of *C. sanguinolenta* showed varying degrees of potency. These tests were performed only on the test organisms that showed inhibition during the antimicrobial screening. With the exception of the chloroform fraction which showed consistent bactericidal results in both MICs and MBCs to all the test organisms, the remaining fractions were bacteriostatic to the microbes. The ethanol extract had MIC values ranging from 8.0 to 32.0 mg/ml for the wild strains while that of the standard strains ranged from 4.0 to 32.0 mg/ml. The chloroform extract had MIC values ranging from 1.0 to 2.0 mg/ml for the standard strains and 0.5 to 2.0 mg/ml for the wild strains with MBC values from 2.0 to 32.0 mg/ml. The petroleum ether fraction exhibited MIC values ranging from 16.0 to 32.0 mg/ml among *P. mirabilis*, *S. saprophyticus* ATCC 15305, *S. aureus* ATCC25923, *P. mirabilis* ATCC 49565 and *E. coli* ATCC 25922, while MIC value of 32.0 mg/ml was observed among *S. saprophyticus* ATCC 15305, *S. aureus* ATCC25923 and *P. mirabilis* ATCC 49565 in the ethyl acetate fraction. The water fraction exhibited MIC values ranging from 8.0 mg/ml to 32.0 mg/ml in twelve of the microbes used (Table 3).

**Table 3:** **The MIC and MBC of the extract and fractions of*C. sanguinolenta***

**Test organisms**	**Ethanolic extract**		**Petroleum ether extract**		**Chloroform extract**		**Ethyl acetate extract**		**Water extract**	
	**MIC**	**MBC**	**MIC**	**MBC**	**MIC**	**MBC**	**MIC**	**MBC**	**MIC**	**MBC**
*Salmonella typhi*	32.0	BST	X	X	1.0	4.0	X	X	8.0	BST
ATCC 19430										
*Salmonella typhimurium* ATCC 14028	X	X	X	X	2.0	4.0	X	X	X	X
*Proteus mirabilis* ATCC 49565	32.0	BST	32.0	BST	2.0	2.0	32.0	BST	16.0	BST
*Pseudomonas aeruginosa* ATCC 27853	X	X	X	X	2.0	4.0	X	X	16.0	BST
*Klebsiella pneumoniae* ATCC 33495	X	X	X	X	4.0	8.0	X	X	X	X
*Escherichia coli*	16.0	BST	16.0	BST	4.0	16.0	X	X	8.0	BST
ATCC 25922										
*Staphylococcus aureus*	4.0	BST	32.0	BST	2.0	4.0	32.0	BST	16.0	BST
ATCC 25923										
*Staphylococcus saprophyticus* ATCC 15305	4.0	BST	16.0	X	2.0	4.0	32.0	BST	32.0	BST
Clinical Isolates										
*Salmonella typhi*	X	X	X	X	1.0	8.0	X	X	X	X
*Salmonella typhimurium*	X	X	X	X	2.0	16.0	X	X	X	X
*Proteus mirabilis*	8.0	BST	32.0	BST	1.0	8.0	X	X	32.0	BST
*Pseudomonas aeruginosa*	32.0	BST	X	X	1.0	8.0	X	X	32.0	BST
*Klebsiella pneumoniae*	32.0	BST	X	X	2.0	32.0	X	X	32.0	BST
*Escherichia coli*	32.0	BST	X	X	1.0	32.0	X	X	32.0	BST
*Staphylococcus aureus*	32.0	BST	X	X	0.5	4.0	X	X	16.0	BST
*Staphylococcus saprophyticus*	32.0	BST	X	X	1.0	4.0	X	X	16.0	BST

X = no test done, BST = Bacteriostatic.

## Discussion

The emergence of antibiotic resistance has prompted scientist to assiduously research into medicinal plants, not only to ascertain claims of efficacy and safety but also to discover alternative candidates for drug development. In the same regard the current study sought to elucidate the antimicrobial activity of ethanolic fractions of *Cryptolepis sanguinolenta*.

Several studies from the West African sub-region have reported the potency of *C*. *sanguinolenta* against clinical malaria [4,15]. The efficacy of its different alkaloids hydroxycryptolepine, cryptolepine HCl and the corresponding base cryptolepine as antifungal, antimalarial and antibacterial has also been reported [16,17].

High resistance observed in the clinical isolates is consistent with emerging antimicrobial resistance worldwide, especially in developing nations like Ghana [18,19]. All bacteria tested were completely resistant to the relatively affordable antibiotics available today; like ampicillin, cloxacillin, tetracycline and penicillin, while observed resistance to gentamicin was below fifty percent. High anti-bacterial resistance to ampicillin, chloramphenicol, streptomycin, sulphonamides and tetracycline in animals and humans has also been reported in Europe and America [20]. The simultaneous use of antimicrobial agents in both human and veterinary medicines have widened the spectrum of resistance to cover trimethoprim, fluoroquinolones and third-generation cephalosporin [21]. Even though there are natural ways microbes develop resistance [22], compliance with pharmaco-vigilance policies will help slow the rate of antibiotic resistance.

The phytochemical analyses revealed the presence of alkaloids, polyuronides and anthocyanosides in the ethanol extract, chloroform and water fractions. However, triterpenes were found only in the water fraction. There are reports on the antimicrobial activities of alkaloids against wide spectra of microbes- according to Cimanga *et al*., [18], ethanol-water extract isolation of the alkaloids quindoline, hydroxycryptolepine, cryptolepine-HCl, and the corresponding base cryptolepine was found to inhibit Gram-positive bacteria and some selected Gram-negative bacteria [18]. Terpenoids are known to have antimicrobial properties [23]. For instance, triterpenes, terpenoids or isoprenoids, are reported to show high antifungal or antimicrobial properties with possible effect on the non-mevalonate pathway. This pathway is essential in fungi, protozoans, gram-negative bacteria and other micro-organisms for the synthesis of cell membrane components, prenylation of proteins and as a secondary source of carbon [24]. Reducing sugars have been reported to have antibacterial property [25,26]. The petroleum ether acting as a defatting agent removed oils from the extract. This may be a contributing factor to the microbial susceptibility observed since most oils from plants have antimicrobial activity [27]. The activity of these phytochemical-constituents may be responsible for the antimicrobial activities observed in the study.

The ethanol extract inhibited both Gram positive and Gram negative bacteria with greater inhibition zones observed among the Gram-positive microbes, probably due to difference in cell wall structure and component. This result is consistent with the work of Mills-Robertson *et al*., [6]. The inhibition of *S. saprophyticus*, *E. coli* and *P. mirabilis* confirm the use of the plant in the treatment of UTI [28]. Aqueous and 80% Ethanol-water extract from the root-bark of *Cryptolepis sanguinolenta* are known to have potent antibacterial, anti-complementary, and moderate antiviral activities, but not antifungal [18]. The petroleum ether fraction of the ethanol extract of *C. sanguinolenta* inhibited the growth of only the standard Gram-positive bacteria; however, both the standard and wild strains of *Proteus mirabilis* were susceptible. *C*. *sanguinolenta* has been shown to contain alkaloids which function to cause cell lysis and morphological changes in *S. aureus*[29]. The inhibition of only the Gram-positive organisms as well as *Proteus mirabilis* by the petroleum ether fraction may suggest the presence of such compounds; hence the fraction may be used as narrow spectrum antimicrobial.

The chloroform fraction registered hundred percent inhibitory activities against all the sixteen isolates. Paulo *et al*., [17] investigated the antimicrobial activity of ethanol extract and five alkaloids of *C. sanguinolenta* and observed that *P. aeruginosa* was resistant to all the alkaloids and the extract. However, this present study observed that the chloroform fraction of *C. sanguinolenta* has inhibitory activity against *P. aeruginosa*; hence, the fraction may be used in the treatment of infections involving these MDR organisms. The chloroform extracts of *Thaumatococcus danielli* leaves have no significant antimicrobial activity against *Salmonella typhimurium, Shigella sp, Escherichia coli* and *Staphylococcus aureus*[30]. Moreover chloroform extracts of *Centrosema pubescens* inhibited the growth of same organisms [31], a justification that the inhibition was not caused by chloroform but rather its constituents. It is thought that chloroform has the potential of extracting some potent compounds which are present in minute proportion in the ethanolic extract. In addition, synergism may be a plausible explanation for this observation [32-34]. These phenomena requires further investigated.

The ethyl acetate fraction inhibited the growth of three out of the sixteen (18.75%). All the other test microbes (n = 13) were not susceptible to the extract. The few organisms inhibited by the fraction could be justified by the dose-dependent activity observed by Cimanga *et al*., [18]. Probably the active compounds contained in the fraction were not sufficient to inhibit most of the organisms. The water fraction of *C*. *sanguinolenta* inhibited twelve out of the sixteen (75.00%) microbes used. The growths of all the Gram-positive microbes were inhibited. Non susceptibility was observed among S*. typhi, S. typhimurium, S. typhimurium* (ATCC 14028) and *K. pneumoniae* (ATCC 33495). The remaining eight Gram-negative organisms were inhibited by the extract. The aqueous fraction has been reported to have a broad spectrum antimicrobial activity [6].

Gram-positive test organisms were generally susceptible to the various fractions used. This may be due to the about 90% peptidoglycan component of the cell wall of Gram-positive bacteria, which is not a regulatory in comparison to the cell membrane of the gram negative bacteria, and therefore, incapable of performing the function of selective permeability, and thus allow substances to sieve through it. The Gram-negative organisms showed varying resistance to all fractions and extract except the chloroform fraction. The Gram-negative bacteria cell wall consist of a peri-plasmic space that contains many hydrolytic enzymes, including β-lactamase, which destroy potentially dangerous foreign substances present in this space.

All the extract and fractions showed MIC values ranging from 0.5 mg/ml to 32 mg/ml but not all were bactericidal. Considering the fact that the chloroform fraction used is a crude extract, the MIC value recorded (0.5 mg/ml) against *S. aureus* and a maximum of 4 mg/ml in the other test organisms shows how potent it could be used as an antimicrobial agent. MICs observed in this study are high compared to some studies like Akiyemi *et al.*[35]. They reported the effectiveness of crude extracts of three medicinal plants from Nigeria against MRSA, and observed MICs ranging from 18.2 to 71.0 ug/ml. Another study [36] determined the antimicrobial activity of three selected plants against Extended Spectrum Beta Lactamase (ESBL)—producing *Escherichia coli* and *Klebsiella pneumoniae* and found majority of the microorganisms inhibited by 40 and 80 *μ*g*/μ*l of the crude extracts. However the current MICs observed is consistently justified by similar studies. Navarro *et al.*[37] investigating 12 methanolic plant extracts normally used in traditional medicine in Mexico to cure infectious diseases, examined the potential antimicrobial activity against *Staphylococcus aureus, Escherichia coli, Pseudomonas aeruginosa* and *Candida albicans.* They reported significant antimicrobial effects, as MICs, ranging between 0.6 and 40 mg/ml of crude extract against the microbes. Another study also reported that Gram-negative bacteria are hardly susceptible to the plant extracts in doses less than 2 × 10^5^ μg/mL [38].

## Conclusion

In conclusion, the study confirmed that fractions of *Cryptolepis sanguinolenta* have significant antibacterial activity. Different fractions have varying antibacterial activity against different organisms. The chloroform fraction had the highest activity, followed by water, ethanol, petroleum ether and ethyl acetate respectively. It is recommended that more research be conducted into the individual compounds in the extracts; there is promise in such to find very low MICs.

## Competing interest

The authors declared that they have no competing interests.

## Authors’ contributions

**FCMR, SCKT, GDE** conceived the study and designed the study protocols and supervised laboratory tests, **WW** collected laboratory specimen, carried out the test and wrote the first draft, **KB** participated in data analysis, and manuscript development. All authors read and approved the manuscript.
